# Role of vitamin D_3_ combined to alginates in preventing acid and oxidative injury in cultured gastric epithelial cells

**DOI:** 10.1186/s12876-016-0543-z

**Published:** 2016-10-07

**Authors:** Francesca Uberti, Claudio Bardelli, Vera Morsanuto, Sabrina Ghirlanda, Claudio Molinari

**Affiliations:** Laboratory of Physiology, Department of Translational Medicine, University of Piemonte Orientale, via Solaroli 17, Novara, 28100 Italy

**Keywords:** Active vitamin D, Gastric acid, Oxidative stress, Gastroprotective drugs

## Abstract

**Background:**

Gastric diseases are a worldwide problem in modern society, as reported in the USA, in the range of 0.5–2 episodes/year/person and an incidence of 5–100 episodes/1000/week according to seasons and age. There is convincing evidence that oxidative stress is involved in the pathogenesis of acute gastric injury. Acid secreted from gastric parietal cells determines mucosal injuries which in turn cause inflammation and oxidative stress. Consequent inflammation produces free radicals by mitochondria thus causing lipid peroxidation, oxidative and acidic stress, which can lead to cell apoptosis. Vitamin D_3,_ the active form of vitamin D, may counteract intracellular cell death and improve epithelial regeneration.

**Methods:**

This study was planned to assess whether vitamin D_3_ is a protective factor against acid injury and oxidative stress in gastric epithelial cells. Primary epithelial cells and GTL-16 cells have been used to test the effects of Grisù® alone or in combination with vitamin D_3_ during oxidative stress or high acid exposition measuring cell viability, ROS production, cellular adhesion time along with apoptotic, autophagic and survival pathways. The combined effect of Grisù® and vitamin D_3_ was found more effective in counteracting the negative consequences of oxidative stress and acidity conditions than some other gastroprotective agents, such as Maalox® or Gaviscon®.

**Results:**

In case of oxidative stress or acidity condition the stimulation with Grisù® alone caused an improvement of cell viability and a reduction of ROS production on epithelial gastric cells. In addition, the adhesion time of the cells was improved. All these effects were increased by the presence of vitamin D_3_. Similar data were also observed in primary gastric epithelial cells confirming the results obtained in GTL-16 cells.

**Conclusions:**

These results suggest that Grisù® in combination with vitamin D_3_ may exert a gastroprotective effect to maintain or restore the integrity of gastric epithelium through an antioxidant pathway, inhibiting apoptosis and activating survival kinases. Moreover, the combination of Grisù® and vitamin D_3_ improves cell viability and decreases ROS production compared to other gastroprotective agents combined with vitamin D_3_. All these data were validated using primary cells isolated from gastric tissue.

**Electronic supplementary material:**

The online version of this article (doi:10.1186/s12876-016-0543-z) contains supplementary material, which is available to authorized users.

## Background

The impact of gastric diseases (for example chronic gastritis, duodenal and gastric ulceration, adenocarcinoma and gastric MALT lymphoma) on human health is a worldwide problem in modern society [1, 2]. For example in the USA, studies have reported gastrointestinal illness rates in the range of 0.5–2 episodes/year/person and incidence of 5–100 episodes/1000/week according to seasons and age. The number of episodes of gastrointestinal illnesses is similar in both 40-year-old studies and in recent ones [3]*.* Human stomach is extremely vulnerable to various attacks; trauma can cause erosion and mucosal epithelium damage which lead to gastrointestinal tract bleeding and/or ulcer perforation and finally worsen the original disease [4]. The gastrointestinal epithelium is a fundamental barrier protecting the gastrointestinal mucosa from damage against the outside environment [4]. The cytoprotective functions against damage may be accomplished in the early phase of epithelial repair known as restitution [5–7], which is the ability of epithelial cells to spread and migrate across the basement membrane to repair the damage. This event is the basis of repair of mucosae after injury and is an important element to grant continuity over broad areas within hours [8, 9]. This reparative event occurs rapidly [10]. The damage to gastric mucosa deriving from stress ulcer has been shown in in vivo models to be possibly repaired within 24 h [11]. Gastric acid (HCl) secreted from gastric parietal cells has been reported to determine gastric mucosal injuries such as peptic ulcer and to induce gastropathy [12]. A prolonged exposition to strong acidic environment causes coagulation necrosis resulting from the desiccating action of the acid on proteins in exposed tissues. A mild gastritis condition is often associated with overindulgence in food and alcohol or stress and each episode causes more lasting damage, eventually resulting in cellular injury which in turn causes inflammation [13]. Consequent inflammation produces free radicals which in turn create even more tissue destruction [14, 15] eventually injuring DNA and potentially leading to stomach cancer, which is one of the most lethal malignancies known so far [16]. In addition, HCl enhances the process of lipid peroxidation in gastric mucosa [17]; the dissipation of mitochondrial transmembrane potential thus induces the production of reactive oxygen species (ROS) by mitochondria causing lipid peroxidation [18, 19]. ROS, including H_2_O_2_, are a major cause of cellular oxidative damage [20] and they play a critical role in the pathogenesis of gastric disorders [2, 21]. Under physiological conditions, gastric epithelium is exposed to high levels of ROS, derived from physical, chemical, or microbiological agents existing in gastric lumen, far higher than in other tissues or biological fluids [22, 23]. Accumulation of intracellular ROS is caused by incomplete reduction of oxygen [24] and this imbalance leads to oxidative stress [24]. H_2_O_2_ is a stable, small and uncharged molecule, that freely diffuses through cell membranes. An excessive level of H_2_O_2_ causes apoptosis, necrosis, and other oxidative damages [25]. When the lower esophageal sphincter is weak, the acid unnaturally moves up into the esophagus, causing gastroesophageal reflux disease (GERD), whose cardinal symptom is heartburn, mainly occurring postprandially.

Therapeutic strategies aim at treating both symptoms and epithelial damage with chemical or mechanical approaches. However, a new molecular approach can be hypothesized in order to counteract intracellular mechanisms leading to cell death and to improve epithelial regeneration.

Recently, a crucial role for vitamin D in digestive system health has been described. Low levels of active form of vitamin D (vitD_3_) are related to poor stomach emptying as well as bloating and constipation or “irritable bowel”. Vitamin D links to several target tissues in the digestive system, in the oral region, epithelial cells of the oral cavity, tongue and gingiva, teeth odontoblast and ameloblast precursor pulp and stratum intermedium cells; in the salivary glands: epithelial cells of striated ducts and granular convoluted tubules, intercalated ducts and acinar cells, as well as myoepithelial cells; in the stomach: neck mucous cells of gastric glands, endocrine cells of the antrum, and muscle cells of the pyloric sphincter; in the small and large intestine: absorptive and crypt epithelial cells; in the pancreas, predominantly islet B-cells [26]. In gastric mucosa, vitamin D appears to have high preventive and therapeutic potentials by stimulating cell proliferation and differentiation [27], and is able to regulate endocrine and paracrine secretion of gastrin with secondary effects, for instance on parietal cell HCl and pepsinogen secreting chief cells [26]. In isolated perfused stomach of vitamin D-deficient rats, gastrin and gastric somatostatin secretion is impaired [27]. Moreover, vitamin D_3_ has been shown to act on smooth muscle cells in pyloric region and in large areas of small intestine [26]. The activity of vitamin D_3_ is mediated by vitamin D receptor (VDR) [28], whose expression has been described in many different tissues including epithelial cells from stomach antrum to duodenum [26]. VDR is a member of the nuclear receptor superfamily which has been shown to be mostly localized in nuclear environment by radiolabeling studies [29], although there is also evidence of their existence in the cytoplasm [30], especially in the absence of ligands and serum in cell culture. Binding its receptor, vitamin D_3_ causes a variety of downstream events, including a protective role against oxidative stress [31], regulation of autophagic pathways [32], and the interplay between apoptosis and survival pathways [31]. In this context vitamin D_3_ may have beneficial effects on gastric health. For this reason, its use could be of greater efficacy in association with other gastroprotective agents like raft-forming alginates, buffers, polysaccharides and biophenols (Grisù®) when compared to other gastroprotectants (e.g. Maalox® or Gaviscon®).

Since VDR/vitD signaling plays a role in growth control of gastric cells, it can be expected that an adequate vitamin D status is required to achieve benefits not only in cancer prevention but also in many other pathologies. Our aim is to demonstrate whether the active form of vitamin D (vitD_3_) can act as a protective factor against oxidative stress in cultured gastric epithelial cells in a mutually supportive manner with other gastroprotective agents.

## Methods

### Cell culture

GTL-16 cell line is a clonal line derived from a poorly differentiated gastric carcinoma cell line [33] and is widely used as a model of gastric epithelial cells. This cell line was donated by the Laboratory of Histology of the University of Eastern Piedmont. Cells were cultured in Dulbecco’s Modified Eagle Medium (DMEM) supplemented with 10 % foetal bovine serum (FBS), 1 % penicillin-streptomycin in incubator at 37 °C, 5 % CO_2_. The cells for the experiments were plated in a different ways; to study cell viability (MTT test) and cell proliferation (crystal violet staining) 1x10^4^ cells were plated on 96 well-plates; to analyze radical oxygen species (ROS) production 1x10^5^ cells were plated on 24 well-plates; to perform immunohistochemistry studies 0.5x10^4^ cells were placed in Culture Slide (BD, Bedford, MA, U.S.A.) with 4 chambers; to analyze the intracellular pathways through Western blot analysis the cells were plated on 60 mm dishes until confluence. Before stimulations the cells were maintained in DMEM without red phenol and FBS and supplemented with 1 % penicillin/streptomycin, 2 mM L-glutamine and 1 mM sodium pyruvate in an incubator at 37 °C, 5 % CO_2_, and 95 % humidity for 18 h. Then the cells were treated with different agents in DMEM without red phenol and supplemented with 0.5 % FBS, 1 % penicillin/streptomycin, 2 mM L-glutamine and 1 mM sodium pyruvate.

### Experimental protocol

The cells were used to study two different biological aspects. In the first set of experiments, the influence of a new alginate preparation (Grisù®) alone or in combination with 100 nM vitD_3_ on cellular adhesion was analyzed by measuring adhesion time through crystal violet assay and vitronectin/fibronectin staining by Western Blot. In this case the agents were used to coat the plates or chamber-slides for 20 min in incubator at 37 °C before seeding the cells. The adhesion time of GTL-16 (a specific number of cells) was checked from 2 min to 720 min (2 min, 5 min, 15 min, 30 min, 60 min, 90 min, 240 min, 720 min) [34]. In the absence of coating, the cells were adherent until 3–5 h.

In the second set of experiments the same agents were added directly in the cells plated for the experimental protocol without coating. Maalox®, Gaviscon® and a proton pump inhibitor (PPI) were tested in the preliminary experiments alone or in combination with 100 nM vitD_3_ to compare the efficacy to Grisù® alone or in combination with 100 nM vitD_3_ on cell viability. In addition, all these agents were also tested in presence of 200 μM H_2_O_2_ or HCl to determine the effects during oxidative stress or gastric acidity. The time of stimulation was from 15 min to 24 h (15 min, 30 min, 60 min, 90 min, 3 h, 24 h). Since the maximum effect was observed after 1 h of treatment this condition was maintained for all successive experiments. Moreover, Maalox®, Gaviscon® and PPI had negative effects on cell viability which were more important in presence of H_2_O_2_ or HCl. In the successive experiments these agents were not tested.

### Agents preparations

Grisù® is a dietary supplement that combines the properties of calcium alginate in a buffer solution, resulting from alkaline salts useful to counteract situations of high acidity, with a tyndalized probiotic (Pylopass®) and an extract of prickly pear and olive leaves (Mucosave®): the extract of prickly pear is useful for its emollient and soothing characteristics at the level of the digestive system.

Grisù®, composed by Mucosave® (0.83 mg/ml), calcium alginate (1.66 mg/ml), magnesium hydroxide (2.66 mg/ml), potassium citrate (4.66 mg/ml) and Pylopass™ (0.66 mg/ml), was dissolved according to solubility reported in manufacturer’s instructions, directly in the DMEM without red phenol and FBS but supplemented with 1 % penicillin/streptomycin, 2 mM L-glutamine and 1 mM sodium pyruvate when used for the adhesion study. Instead for the second set of experiments Grisù®, Maalox®, Gaviscon® and PPI were dissolved directly in the medium used for stimulations respecting the solubility reported in manufacturer’s instructions. In both sets of experiments, the solutions were used without dilutions. The active form of vitamin D (1,25-dihydroxyvitamin D_3_, catalog number: D1530, Sigma-Aldirch, Milan, Italy) was prepared in absolute ethanol at 10^-3^ M and used at final concentration of 100 nM prepared directly in medium. The percentage of ethanol was less than 0.001 %. HCl was used to prepare an acidified medium (pH 4) and added to the cells without dilution.

### MTT test

At the end of each stimulation, MTT-based In Vitro Toxicology Assay Kit (Sigma-Aldrich) was used to determine cell viability, as previously described [31]. Cells were incubated with 1 % MTT dye for 2 h at 37 °C in incubator, and then, the purple formazan crystals were dissolved in equal volume of MTT Solubilization Solution. Cell viability was determined by measuring the absorbance through a spectrometer (VICTORX3 Multilabel Plate Reader) at 570 nm with correction at 690 nm, and calculated by comparing results to control cells (100 % viable).

### Crystal violet staining

The cells treated with Grisù® alone or in combination with vitD_3_ were also analyzed by crystal violet staining to study adhesion. GTL-16 cells at the end of each time-point were fixed with 1 % glutaraldehyde for 15 min at RT, washed and stained with 100 μl of 0.1 % crystal violet for 20 min at RT. The multi-well plates were rinsed, dry and the images of adherent aqueous cells were taken and analyzed by ImageJ software. To obtain cell number estimation 100 μl of 10 % acetic acid were added and mixed before reading the absorbance at 595 nm through a spectrometer (VICTORX3 Multilabel Plate Reader). The number estimation was calculated by comparing results to the control cells (Control T0).

### ROS production

The rate of superoxide anion release was measured using a standard protocol [31] and determined as a superoxide dismutase-inhibitable reduction of cytochrome C. In both treated and untreated cells, 100 μl of cytochrome C were added, and in another sample, 100 μL of superoxide dismutase were also added for 30 min in incubator (all substances from Sigma-Aldrich). The absorbance was measured at 550 nm in a spectrometer (VICTORX3 Multilabel Plate Reader) and the O_2_ was expressed as means ± SD% of nanomoles per reduced cytochrome C per microgram of protein compared to control [35].

#### Western Blot

GTL-16 cells were lysed in iced Complete Tablet Buffer (Roche) supplemented with 2 mM sodium orthovanadate and 35 μg of proteins resolved on 15 or 5 % SDS-PAGE gels. Polyvinylidene fluoride membranes (PVDF, GE Healthcare Europe GmbH, Milan, Italy) were incubated overnight at 4 °C with specific primary antibody: anti-phospho-ERK^Thr202/Tyr204^ (1:1000, Cell-SIgnaling), anti-VDR (1:250, Santa-Cruz), anti-Annexin V (1:1000, Sigma), anti-Beclin1 (1:250, Santa-Cruz), anti-Caspase 8 (1:400, Sigma), anti-Bax (1:200, Santa-Cruz), anti-Vitronectin (1:250, Santa-Cruz) and anti-Fibronectin (1:250, Abcam, UK). The protein expressions were normalized and verified through β-actin detection (1:5000; Sigma, Milan, Italy).

### Immunocytochemistry

The cells cultured in chamber slides were fixed using an iced fixative solution (3.7 % formaldehyde, 3 % sucrose in PBS 1X) for 20 min at RT, permeabilized with ice 0.5 % Triton X-100 in PBS 1X in ice at 4 °C for 20 min, incubated with 3 % hydrogen peroxide in PBS 1X for 8 min and then maintained in a blocking solution (PBS 1X with 3 % albumin from bovine serum-BSA, Sigma, Milan, Italy) for 1 h in at RT, as previously described [36]. After this time the slides were incubated overnight at 4 °C in a humidified chamber with specific primary antibody VDR receptor (1:50). Then chamber slides were incubated before with diluted biotinylated secondary antibody solution (Dako Italia, Milan, Italy), then with VECTASTAIN® ABC Reagent (Dako Italia, Milan, Italy), and finally in peroxidase substrate solution (Peroxidase/DAB, Dako Italia, Milan, Italy), and counterstained with Mayer’s hematoxylin. The number of positive cells was calculated as described elsewhere [37]; briefly, 12 different areas (1 mm^2^) randomly selected from each section were taken, and the number of signals was determined using ImagePro 3 software (NIH, Bethesda, US). The results are expressed as means ± SD (%).

### Statistical analysis

Results are expressed as means ± SD of at least 5 biological replicates for each experimental protocol and each replicate was reproduced 3 times. One-way ANOVA followed by Bonferroni post hoc test was used for statistical analysis. Mann-Whitney U test was used to compare percentages of responses. *P*-value <0.05 was considered statistically significant.

## Results

### *Influence of Grisù*® *alone or in combination with vitD*_*3*_*on adhesion capacity*

**Fig. 1:**
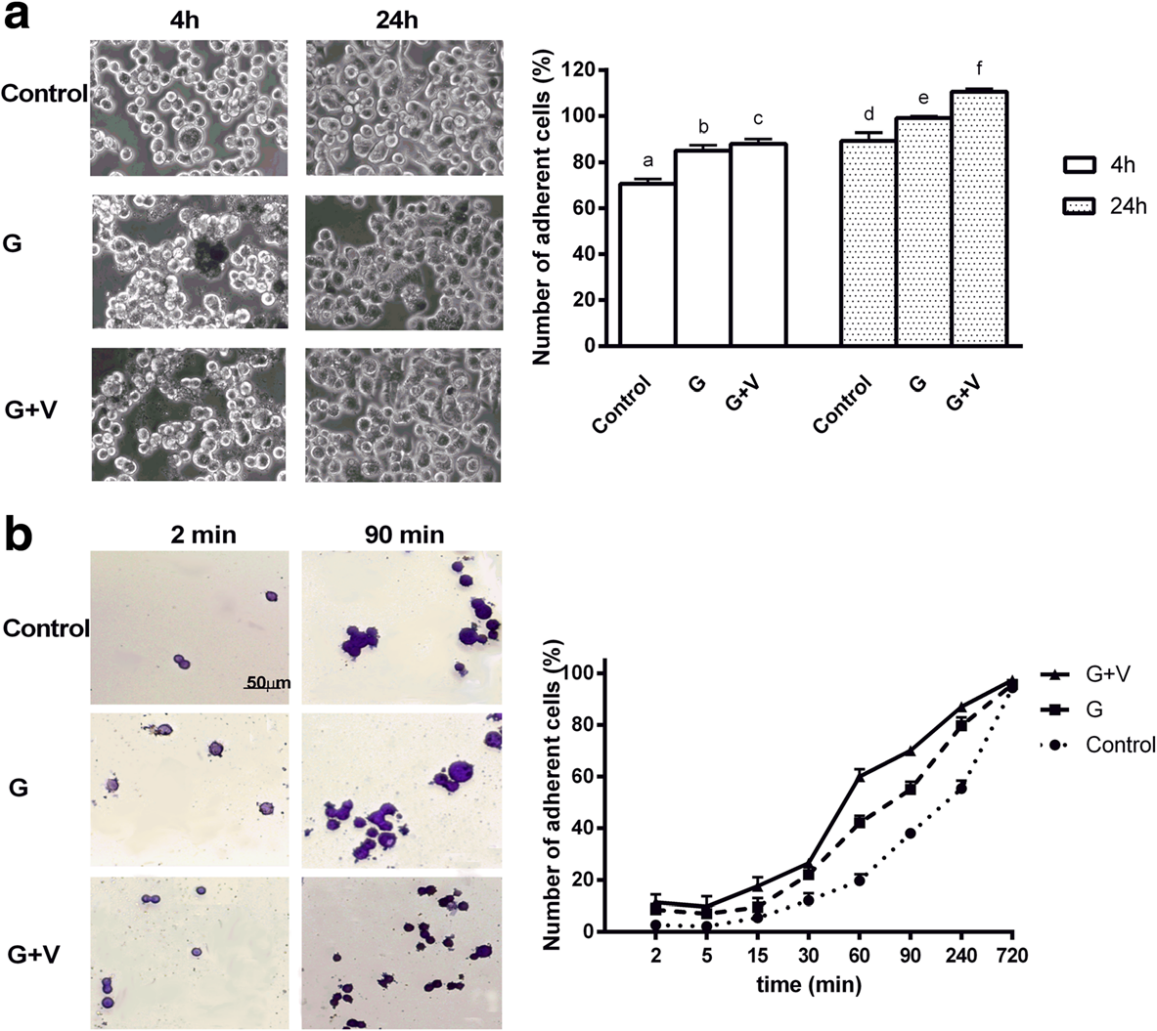
Adhesion time analysis of GTL-16. In **a** on the left, optical microscopy images of adherent GTL-16 taken at original magnification 20X. On the right, the number of adherent cells for each treatment is reported. The ratio reports a mean ± (SD) (%) of adherent cells counted in 12 different areas. Data reported are means of 5 biological replicates. G = Grisù®; G + V = co-stimulation with Grisù® and vitD_3_. b, c, d *p* < 0.05 *vs* a; a, f, e *p* < 0.05 *vs* d; e *p* < 0.05 *vs* b; f *p* < 0.05 *vs* c. In **b** on the left, optical microscopy images of the cells treated with Grisù® alone or in combination with vitD_3_ after 2 min and 90 min are shown. The scale reported in the first picture is to be considered valid for all images. On the right, the cell counting of adherent cells during time (at 2 min, 5 min, 15 min, 30 min, 60 min, 90 min, 240 min, 720 min) is reported. The ratio reports a mean ± (SD) (%) of adherent cells counted in 12 different areas of 5 biological replicates. The abbreviations are the same reported in **a**. All samples are significant *p* < 0.05 *vs* control

**Fig. 2:**
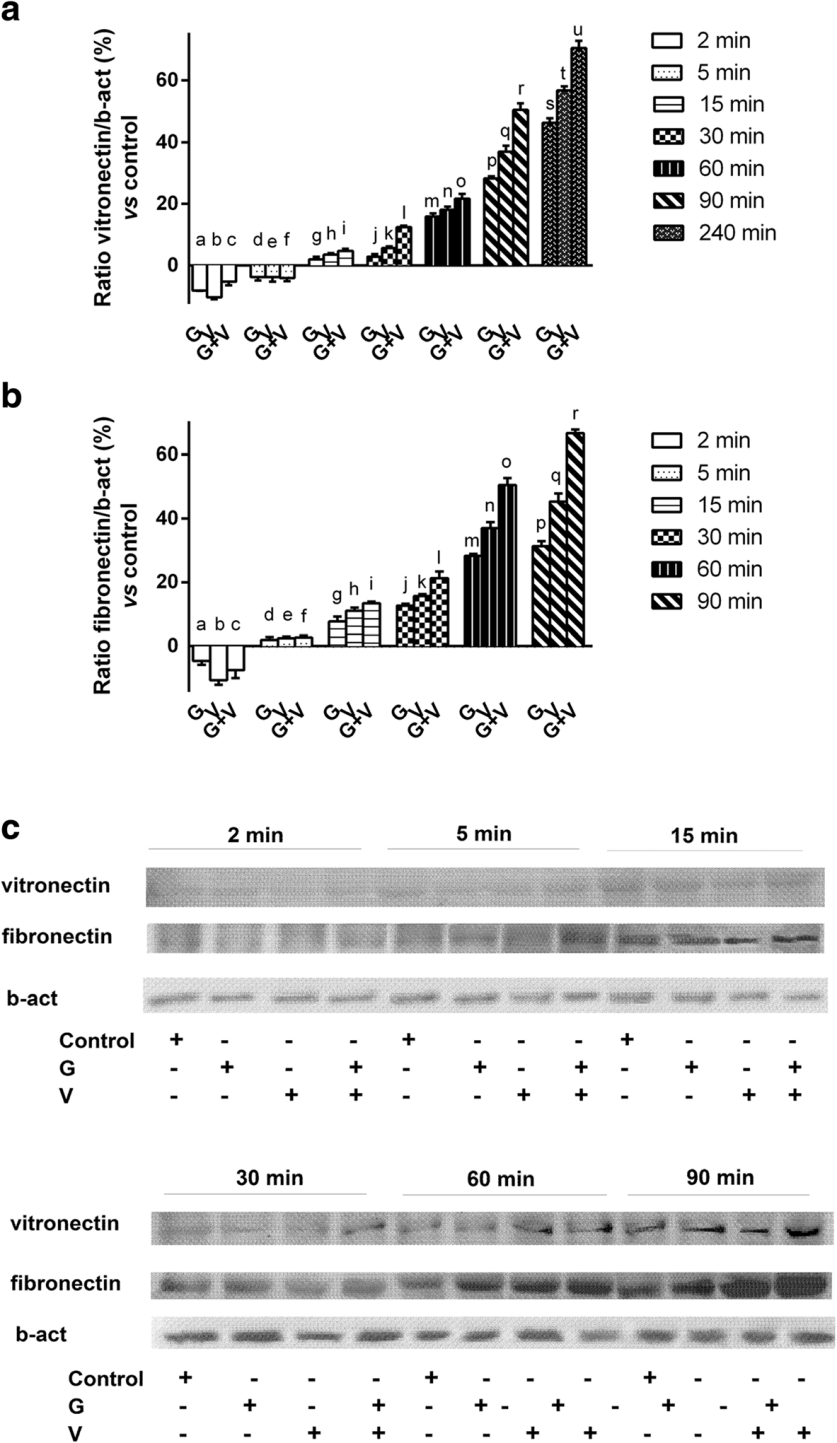
Western blot and densitometric analysis of vitronectin and fibronectin. G = Grisù®; V = vitD_3_; G + V = Grisù® combined with vitD_3_. The ratio reports a mean ± (SD) (%) of 5 biological replicates normalized to control values (line 0 %). In **a** densitometric analysis of vitronectin normalized to control values during time (from 2 to 240 min). a, b, c, d, e, f, h, i, j, k, l, m, n, o, p, q, r, s, t, u *p* < 0.05 *vs* control; a, b *p* < 0.05 *vs* c; e *p* < 0.05 *vs* f; i *p* < 0.05 *vs* g; j, k *p* < 0.05 *vs* l; m, n, *p* < 0.05 *vs* o; p, q *p* < 0.05 vs r; s, t *p* < 0.05 *vs* u. In **b** densitometric analysis of fibronectin normalized to control values during time (from 2 to 90 min). d, e, f, g, h, i, j, k, l, m, n, o, p, q, r *p* < 0.05 *vs* control; g, h *p* < 0.05 *vs* i; j, k *p* < 0.05 *vs* l; m, n *p* < 0.05 *vs* o; p, q *p* < 0.05 *vs* r. In **c** an example of Western blot of protein extracts analyzed by immunoblotting with specific antibodies against the indicated proteins

Grisù® acts as a physical barrier for gastric epithelial cells and protects against hyperacidity. The first set of experiments was performed because of a difference in cell adhesion after 4 h in the sample treated with Grisù® (85.17 ± 2.25 %) compared to control (70.63 ± 2.12 %, *p* < 0.05) (Fig. 1a). This augmented number of adherent cells was more evident after 24 h (99.2 ± 0.72 % compared to control 89.33 ± 3.51 %). The presence of vitD_3_ improves this effect (at 4 h 88.07 ± 2.1 %; at 24 h 110.6 ± 1.25 %). Since this time is more suitable to explain the influence of Grisù®, the time-point was changed and Grisù® alone or in combination with vitD_3_ was used to coat the plates. As reported in Fig. 1b and Table 1, in crystal violet staining, the number of adherent GTL-16 cells was time-dependent and in presence of Grisù® alone significantly increased (*p* < 0.05) starting from 2 min after seeding (8.5 ± 2.12 % compared to control 2.67 ± 1.63 %). This increase was enhanced in presence of vitD_3_ (of about 33 % compared to Grisù® alone, *p* < 0.05). All these effects were more evident in the course of time (*p* < 0.05) and after 12 h almost all of the cells were attached (*p* < 0.05). Vitronectin and Fibronectin, two extracellular matrix glycoproteins well-known to be involved in adhesion, were analyzed by Western Blot, to explain the mechanism involved. As reported in Fig. 2a and c, Vitronectin expression assessed by Western blot and densitometric analysis, showed an increase in a time-dependent manner and the presence of Grisù® starting from 15 min was able to improve this expression (1.93 ± 0.9 %, *p* < 0.05) compared to control. The presence of co-stimulation with vitD_3_ was able to amplify the effect of Grisù® alone starting from 15 min (4.69 ± 0.65 %) and this amplified effect was maintained for the entire test time, being evident at 90 min (*p* < 0.05) compared to Grisù® alone (of about 78 %). The effect of vitD_3_ alone was also analyzed, but did not show a significant increase on vitronectin expression up to 60 min (18.06 ± 1 % compared to control), thus showing the significant ability of vitD_3_ to improve the effect of Grisù®. The administration of Grisù® combined with vitD_3_ after 4 h treatment was able to improve the Vitronectin expression according to the data observed in crystal violet staining (*p* < 0.05).

**Table 1:** Cell counting of crystal violet staining

Time (min)	Control	G	G + V
2	2.67 ± 1.63	8.5 ± 2.12 ^a^	11.33 ± 3.05 ^a^
5	2.17 ± 0.98	7 ± 2.83	9.67 ± 4.04 ^a^
15	5.5 ± 2.38	9.5 ± 3.54	17.5 ± 3.54 ^a^
30	12 ± 2.83	22 ± 2.83 ^a^	26.5 ± 0.71 ^a^
60	19.67 ± 2.52	42 ± 2.84 ^a^	60 ± 2.83 ^a^
90	38 ± 2	55 ± 3 ^a^	70 ± 2 ^a^
240	55.33 ± 3.05	79.67 ± 3.215 ^a^	87 ± 2 ^a^
720	94.33 ± 2.082	95.68 ± 1.528	97.33 ± 1.15

Data are shown as a mean ± (SD) (%) of adherent cells counted in 12 different areas of 4 technical replicates. ^a^ *p* < 0.05 vs control. The abbreviations are the same described in figure legends

Similar time-dependent effects were also observed in Fibronectin expression, in GTL-16 treated with Grisù® alone or in combination with vitD_3_, confirming the role of vitD_3_ in modulating the capacity of Grisù®. These data obtained by Western Blot and densitometric analysis are shown in Fig. 2b and c.

### *Mechanisms induced on cultured GTL-16 cells by Grisù*® *alone or in combination with vitD*_*3*_

**Fig. 3:**
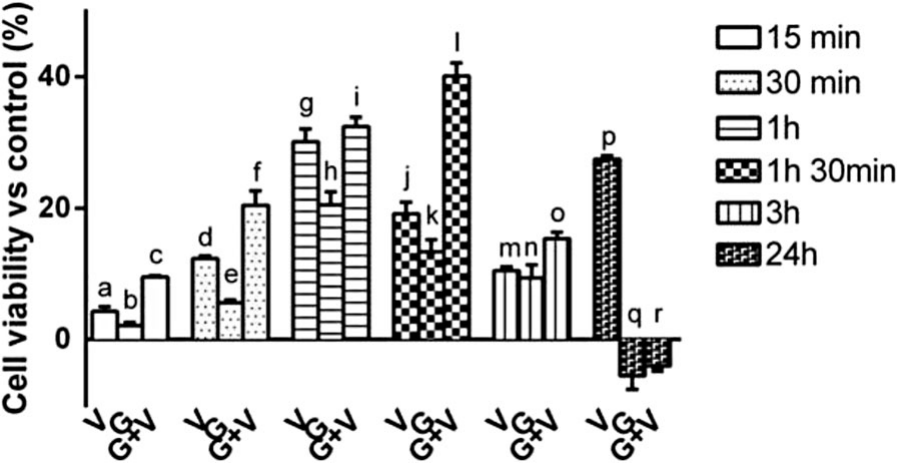
Time course of cell viability in GTL-16. The effects of vitD_3_ (V), Grisù® (G) and Grisù® combined with vitD_3_ (G + V) *vs* control (line 0 %) from 15 min to 24 h are reported. Data represent as a mean ± (SD) (%) of 6 biological replicates. a, b, c, d, e, f, g, j, k, l, m, n, o, p; *p* < 0.05 *vs* control; a, b *p* < 0.05 *vs* c; c, d, e *p* < 0.05 *vs* f; c, f, h *p* < 0.05 *vs* i; i, j, k, o, r *p* < 0.05 *vs* l; n *p* < 0.05 *vs* o; o, p *p* < 0.05 *vs* r

MTT test was performed on a time-course study to determine the best stimulation time for the influence of Grisù® alone or in presence of 100 nM vitD_3_ on cell viability. As reported in Fig. 3, the maximum effect of Grisù® alone was observed after 1 h of stimulation (20.55 ± 1.99 %, *p* < 0.05) and in presence of vitD_3_ this effect was amplified of about 58 %, (*p* < 0.05). For this reason, this time of stimulation was maintained for all successive experiments.

**Fig. 4:**
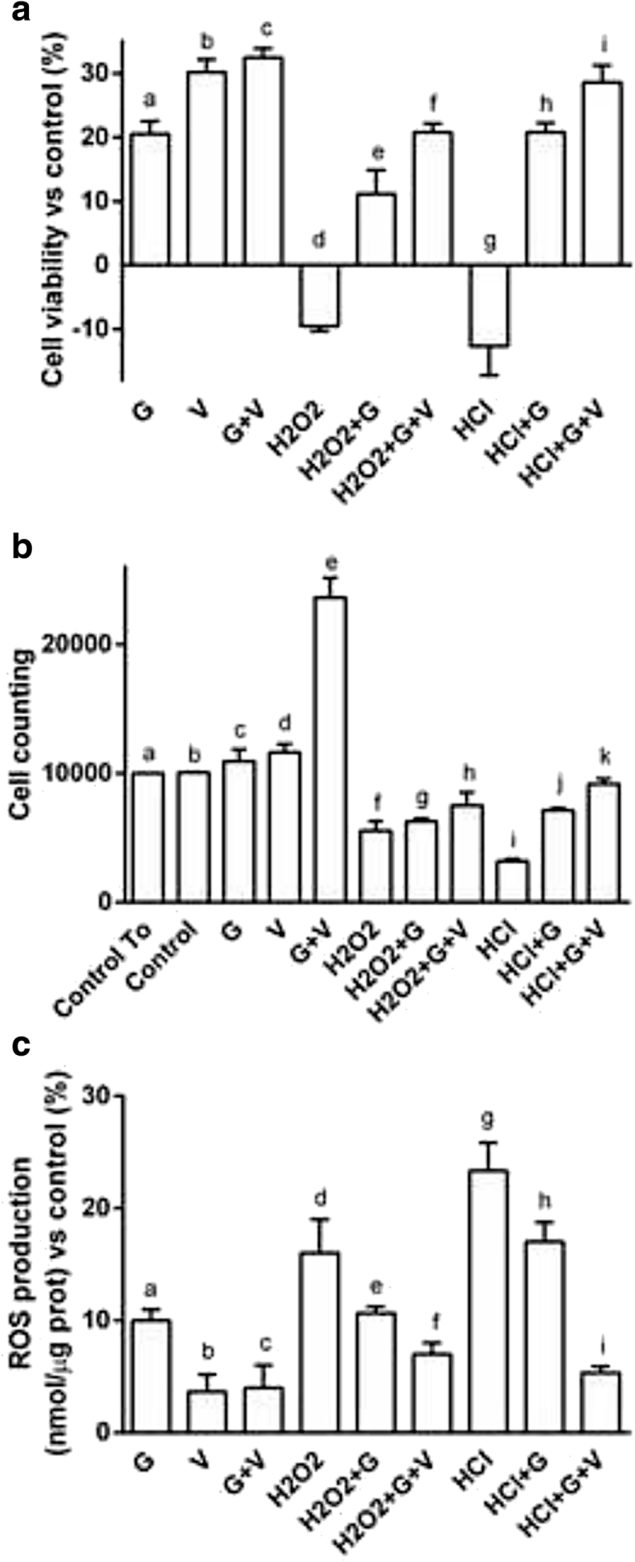
Effects of Grisù® alone or in combination with vitD_3_ during acidity and oxidative stress. G = Grisù®; V = vitD_3_; G + V = Grisù® combined with vitD_3_. Data represent a mean ± (SD) (%) of 6 biological replicates. In **a** cell viability: a, b, c, d, e, f, g, h, i *p* < 0.05 *vs* control (line 0 %); c *p* < 0.05 *vs* a; e, f *p* < 0.05 *vs* d; f *p* < 0.05 *vs* e; h, i *p* < 0.05 *vs* g; i *p* < 0.05 *vs* h; i *p* < 0.05 *vs* f. In **b** cell counting: c, d, e, f, g, h, i, j *p* < 0.05 *vs* control; e *p* < 0.05 *vs* c; e *p* < 0.05 *vs* d; h *p* < 0.05 *vs* g; h *p* < 0.05 *vs* f; k, j *p* < 0.05 *vs* i; k *p* < 0.05 *vs* j; k *p* < 0.05 *vs* h. In **c** ROS production: a, c, d, e, f, g, h, i, *p* < 0.05 *vs* control (line 0 %); c, b *p* < 0.05 *vs* a; f, e *p* < 0.05 *vs* d; f *p* < 0.05 *vs* e; i, k *p* < 0.05 *vs* g; i *p* < 0.05 *vs* h

**Fig. 5:**
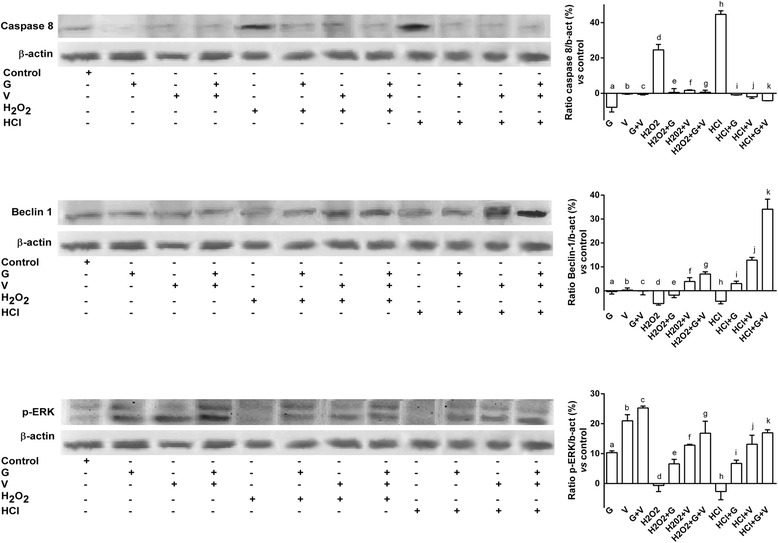
Western blot and densitometric analysis of apoptotic, autophagic and survival pathways. The images and data reported are obtained from 5 biological replicates. G = Grisù®; V = vitD_3_; G + V = Grisù® combined with vitD_3_. Densitometric analysis are expressed as means ± (SD) (%) normalized to control values (defined as 0 %). Caspase 8 *p* < 0.05: a, d, h, k *vs* control; e, f, g *vs* d; g *vs* k; f, j *vs* b; a, k *vs* c; i, j, k *vs* h; c, i, j *vs* k; e, j *vs* a. Beclin *p* < 0.05: d, f, g, h, j, k *vs* control; g, k *vs* c; e, f, g *vs* d; e, k *vs* g; f, j *vs* b; i, j, k *vs* h; i, j *vs* k; i *vs* a. ERK: a, b, c, e, f, g, i. j, k *vs* control; e *vs* g; i *vs* k; a, g, k *vs* c; e, f, g *vs* d; i, j, k *vs* h; f, j *vs* b; e, i *vs* a

In order to clarify the efficacy of Grisù® alone and in combination with 100 nM vitD_3_ on cell viability, on cell proliferation and on ROS production in gastroprotection, all the following experiments were performed in presence or absence of pretreatment for 30 min with 200 μM H_2_O_2_ or HCl. As reported in Fig. 4 the pre-treatment for 30 min with 200 μM H_2_O_2_ or HCl caused a significant reduction (*p* < 0.05) of cell viability and cell proliferation and increased ROS production (*p* < 0.05) compared to control. Grisù® alone was able to counteract the negative effects exerted by H_2_O_2_ or HCl on cell viability, cell proliferation and ROS production (*p* < 0.05); in particular, the most evident beneficial effects were observed in reversing the influence exerted by HCl. In presence of the combination with 100 nM vitD_3_ the beneficial effects were amplified (*p* < 0.05) maintaining the efficacy in counteracting the influence of HCl. The apoptotic (in particular Annexin V, Bax, Caspase 8), autophagic (Beclin 1) and survival (ERK/MAPK) pathways were investigated to explain the intracellular mechanisms involved in the protective actions induced by Grisù® alone or in combination with 100 nM vitD_3_. In addition, the VDR receptor was analyzed to study the role of vitD_3_ in all these effects. Western blot and densitometric analysis (Fig. 5 and Additional file 1) showed a significant increase of Annexin V, Bax and caspase-8 expressions (*p* < 0.05) compared to control in presence of pretreatment with 200 μM hH_2_O_2_ or HCl alone and the maximum effects were observed with HCl. On the contrary the markers of autophagy and survival pathways were maintained at a basal level. Either Grisù® or vitD_3_ alone caused an increase in ERK1/2 and VDR expression compared to control (*p* < 0.05). The combination of Grisù® and vitD_3_ amplified (*p* < 0.05) the effects on ERK1/2 and VDR expressions (25.26 ± 0.64 % and 39.12 ± 3.46 %, respectively) compared to Grisù® (10.31 ± 0.59 % and 0.33 ± 1.15 %, respectively) or vitD_3_ alone (20.96 ± 2.05 % and 29.48 ± 1.34 %, respectively). The same stimulations performed after the treatments with 200 μM H_2_O_2_ or HCl, were able to reverse the negative effects acting on the expression of ERK1/2 and Beclin 1; in particular the combination of Grisù® and vitD_3_ was able to amplify the activation of autophagic and survival pathways. In addition, this combination was able to improve the reduction of the apoptotic marker, such as Annexin V, Bax and caspase 8.

**Fig. 6:**
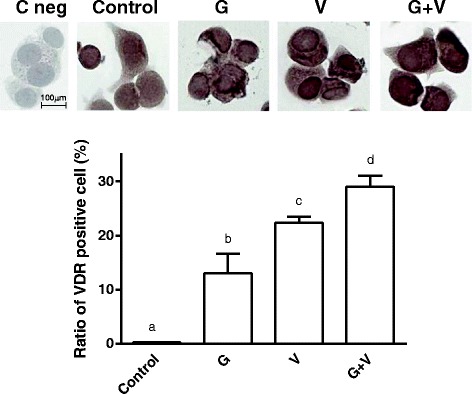
Study of VDR by immunocytochemistry staining. G = Grisù®; V = vitD_3_; G + V = co-stimulation with Grisù® and vitD_3_. b, c, d *p* < 0.05 *vs* control; b, c *p* < 0.05 *vs* d. The images were taken by optical microscopy at original magnification 20X. Data reported are expressed as means ± (SD) (%) of 5 biological replicates

Finally, the involvement of VDR in the beneficial effects above described was also confirmed by immunocytochemical staining (Fig. 6) and by Western Blot analysis (Additional file 1).

**Fig. 7:**
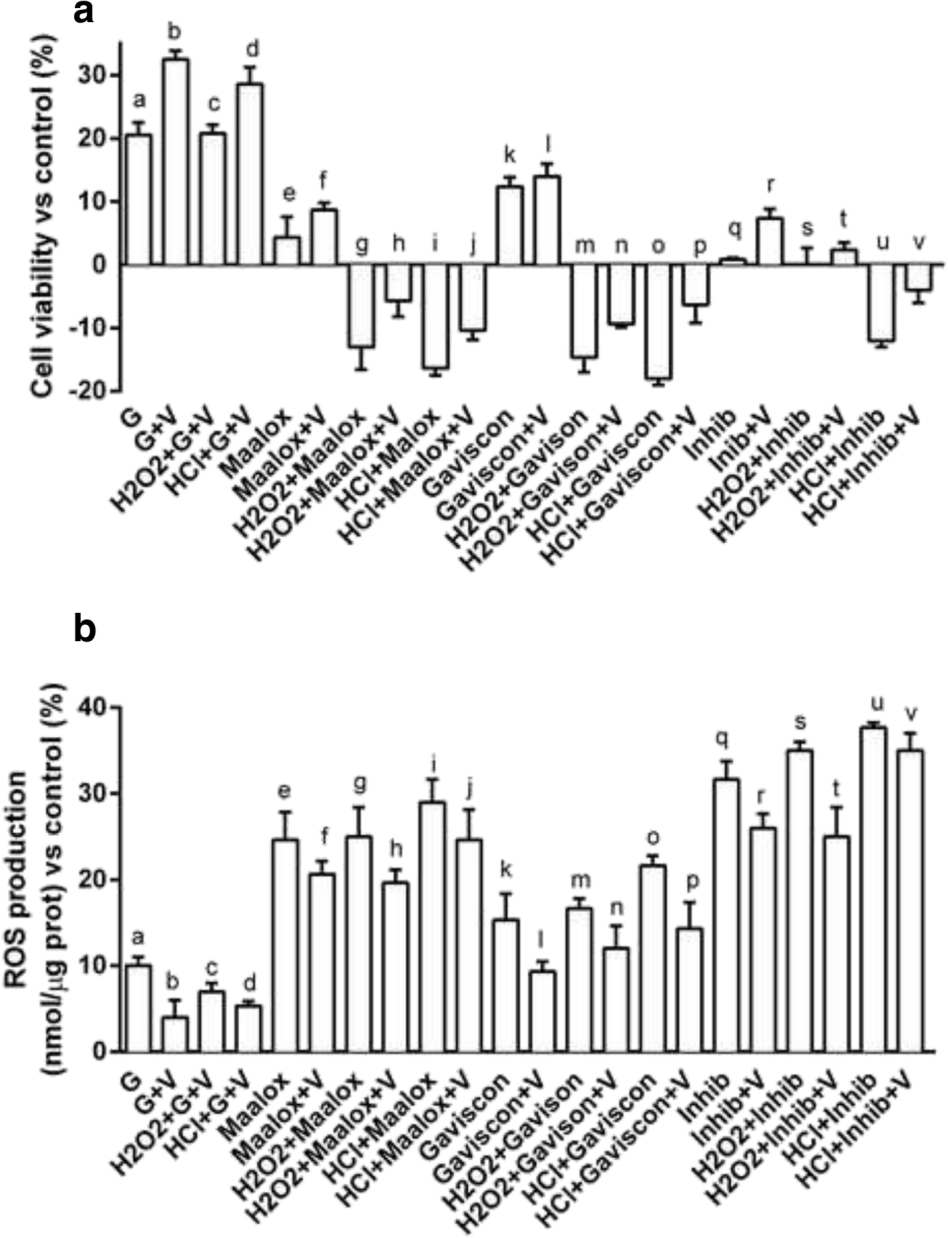
Cell viability and ROS production in GTL-16 treated with other gastroprotectants with vitD_3_. G = Grisù®; V = vitD_3_; G + V = co-stimulation with Grisù® and vitD_3_. Data reported are expressed as means ± (SD) (%) of 5 biological replicates. In **a**: a, b, c, d, f, g, h, i, j, k, l, m, n, o, p, r, u, v *p* < 0.05 *vs* control (line 0 %); b, d, e, f, g, h, i, j, k, l, m, n, o, p, q, r, s, t, u, v *p* < 0.05 *vs* a; a, c, e, f, g, h, i, j, k, l, m, n, o, p, q, r, s, t, u, v *p* < 0.05 *vs* b; g, h, m, n, s, t *p* < 0.05 *vs* c; i, j, o, p, u, v *p* < 0.05 *vs* d; g, h, i, j *p* < 0.05 *vs* e; h, j, *p* < 0.05 *vs* f; m, n, o, p *p* < 0.05 *vs* k; n, p *p* < 0.05 *vs* l; r, u, v *p* < 0.05 *vs* q. In **b**: a, c, e, f, g, h, i, j, k, l, m, n, o, p, q, r, s, t, u, v *p* < 0.05 *vs* control (line 0 %); b, e, f, g, h, i, j, m, o, q, r, s, t, u, v *p* < 0.05 *vs* a; e, f, g, h, i, j, k, m, n, o, p, q, r, s, t, u, v *p* < 0.05 *vs* b; g, h, m, s, t *p* < 0.05 *vs* c; i, j, o, p, u, v *p* < 0.05 *vs* d; o *p* < 0.05 *vs* k; t, u *p* < 0.05 *vs* q; v *p* < 0.05 *vs* r

The effects of combination of Grisù® and vitD_3_ were compared to other gastroprotective agents on cell viability and ROS production, to confirm their ability to protect the epithelial gastric cells from oxidative stress or acidity injury. As reported in Fig. 7, the combination of Grisù® with vitD_3_ was able to induce the maximal beneficial effects on cell viability and ROS production compared to other gastroprotective agents alone (Maalox®, Gaviscon® or PPI, *p* < 0.05). In addition, in presence of oxidative stress caused by H_2_O_2_ or HCl acidified medium, the combination of Grisù® with vitD_3_ was able to positively modulate these negative conditions and significantly reduce ROS production. This effect was significantly better if compared to Maalox®, Gaviscon® and PPI.

In some additional tests, vitD_3_ was added to other gastroprotective agents (Maalox®, Gaviscon® and PPI) and the effects have been compared to those induced by Grisù® combined with vitD_3_. As shown in Fig. 7, Grisù® with vitD_3_ showed greater effectiveness to counteract the negative effects of oxidative stress and acidity condition on cell viability and ROS release compared to other gastroprotectants.

Similar data were also observed in primary gastric cell cultures, where Grisù® with vitD_3_ was able to counteract more effectively than other gastroprotectants, the negative effects of oxidative and acidic stress.

### Validation in primary epithelial cells

Further experiments were carried out in primary gastric epithelial cells, in order to confirm previously obtained results about the protective effects exerted by Grisù® alone or combined with vitD_3_ during oxidative and acidity injury. Method used to isolate the primary cell cultures is described in Additional file 2. Other methods were the same used in GTL-16 cells experiments. Some results on primary gastric cells were reported in Additional file 2. Particularly, the improvement of adhesion properties induced by Grisù® combined with vitD_3_; the beneficial effects on cell viability and ROS production in counteracting H_2_O_2_ or HCl oxidative injury; the involvement of survival kinase in preventing apoptosis, are shown.

**Fig. 8:**
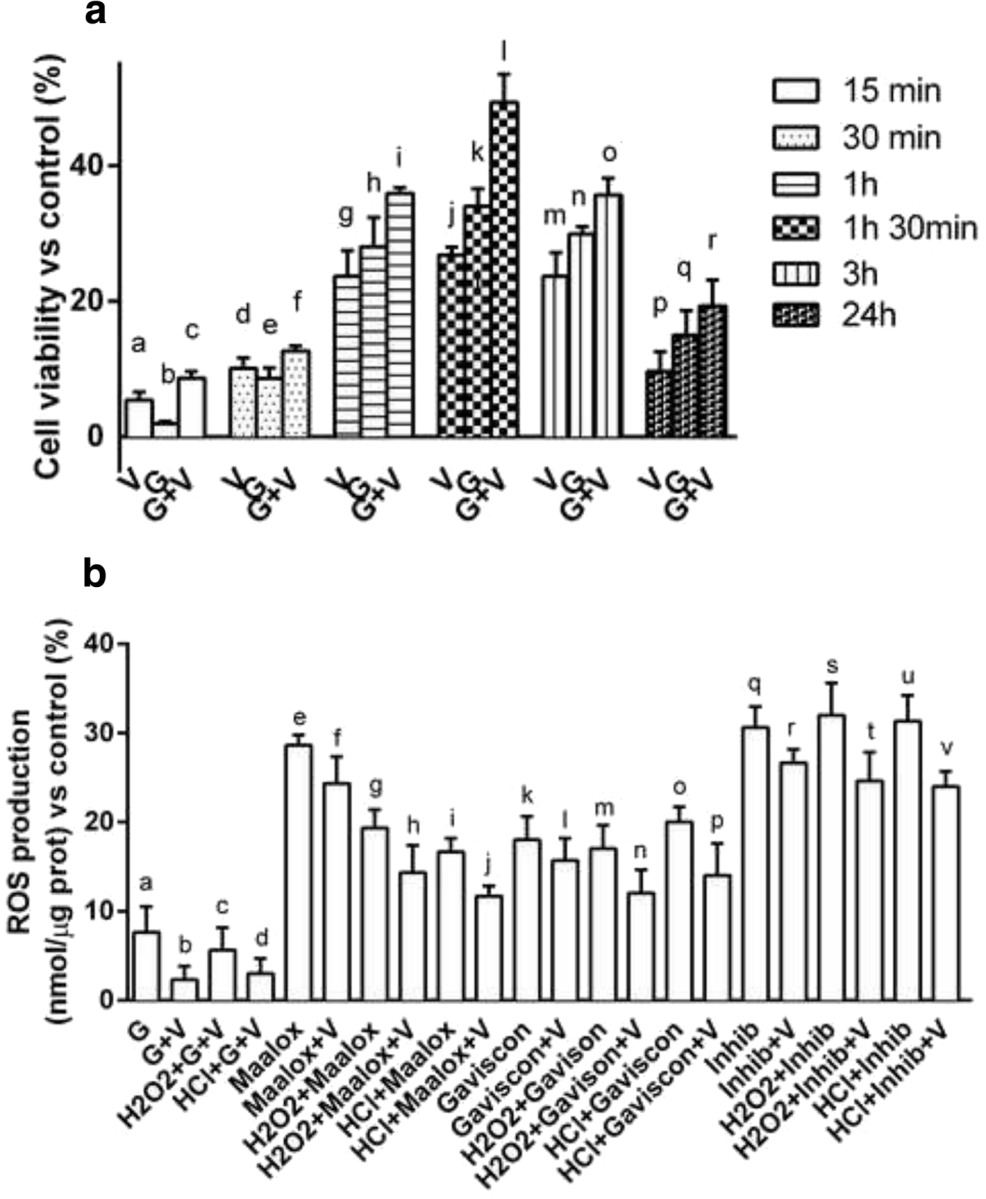
Time-course of cell viability and ROS production in primary gastric cells. In **a** primary gastric cells treated with Grisù® alone or combined with vitD_3_ analyzed at 15 min, 30 min, 1 h, 1 h 30 min, 3 h, and 24 h. V = vitD_3_; G = Grisù®; G + V = Grisù® combined with vitD_3_. Data are reported as means ± (SD) (%) of 3 biological replicates. a, c, d, e, f, g, h, i, j, k, l, m, n, o, p, q, r *p* < 0.05 *vs* control (line 0 %); c *p* < 0.05 *vs* b; g, h *p* < 0.05 *vs* i; j, k *p* < 0.05 *vs* l; m *p* < 0.05 *vs* o; p *p* < 0.05 *vs* r. In **b** the effect on ROS production of Grisù® combined with vitD_3_ was compared to the effects of different gastroprotectants alone and with vitD_3_. The results are expressed as means ± (SD) (%) of 3 biological replicates. G = Grisù®; V = vitD_3_; G + V = Grisù® combined with vitD_3_. a, e, f, g, h, i, j, k, l, m, n, o, p, q, r, s, t, u, v *p* < 0.05 *vs* control (line 0 %); e, f, g, h, i, k, l, m, o, p, q, r, s, t, u, v *p* < 0.05 *vs* a; e, f, g, h, i, j, k, l, m, n, o, p, q, r, s, t, u, v *p* < 0.05 *vs* b; g, h, m, n, t *p* < 0.05 *vs* c; i, j, o, p, u, v *p* < 0.05 *vs* d; g, h, i, j *p* < 0.05 *vs* e; h, j *p* < 0.05 *vs* f; v *p* < 0.05 *vs* q

Here are reported two of most important effects of Grisù alone and combined with vitD_3_ on cell viability and ROS production. As reported in Fig. 8a the time-course study confirms that Grisù® alone and Grisù® combined with vitD_3_ are able to enhance cell viability in similar manner to what observed in GTL-16. The maximum effect was observed after 1 h 30 min (*p* < 0.05). So, time of stimulation was maintained for all subsequent experiments. Finally, in primary gastric cells have been also confirmed the efficacy of Grisù® alone and combined with vitD_3_ compared to others gastroprotectants to prevent the damage induced by oxidative stress and acidity. As reported in Fig. 8b, vitD_3_ can modulate the negative effects on ROS production only in presence of Grisù®. This effect was also observed in GTL-16 cells. Therefore, it can be hypothesized that Grisù® could act like a substrate capable of promoting the effects of vitD_3_.

## Discussion

The integrity of gastrointestinal epithelium and mucosa is known to be maintained by a number of secreted factors [38] and mediated by rapid proliferation and migration of mucosal epithelial cells [4] to resurface epithelial defects after various forms of mucosal epithelium injury [4] and to establish a barrier against the stress inducers. As reported in literature in several in vitro and in vivo studies, these factors protect gastric mucosal epithelium from a variety of noxious agents, including bacterial toxins, chemicals and drugs [4]. Epithelial migration, which is a fundamental phase in the healing process after gastric ulcer, is characterized by complex alterations in adhesion between cells and the extracellular matrix [39]. In this study the effects of vitD_3_ on an in vitro experimental model of gastric cells have been observed. Findings of this work demonstrate that vitD_3_ can exert beneficial effect on gastric cells in terms of improved viability and adhesivity or reduced ROS production. Moreover, this study permits to demonstrate that Grisù® alone is able to improve the adhesivity of cells, reducing the time of action (starting from 2 min from seeding, also measured by cell counting) but this effect is amplified by the presence of vitD_3_. Moreover, we have found out that this effect is crucial on cells migration and that two important extracellular matrix glycoproteins, Vitronectin and Fibronectin, are involved in this effect mediated by Grisù® both alone and in presence of vitD_3_. This consideration is even more important because it indicates that vitD_3_ is able to improve the beneficial effects induced by alginates, thus supporting data about the mechanism of gastroprotection induced by alginates [40]. This hypothesis is also supported by other in vitro studies where VDR activation has been shown not only to enhance intracellular junctions but also to promote mucosal wound repair and to mediate the activity of vitD_3_ thus inducing junction proteins expression [41]. As observed by Miyazaki et al., the reparative events following acute gastric injury occur rapidly [10]. There is evidence that oxidative stress plays an important role in the pathogenesis of acute gastric injury [42–44]. For example, the role of oxidative stress in gastrointestinal pathogenesis was first described in acute intestinal injury in ischemia/reperfusion models [42]. Following studies suggested that ROS are generally involved in acute injury of the gastrointestinal tract [43, 44]. It is noteworthy that experimental evidence shows how ROS production or superoxide (O_2_^-^) are induced both by cellular exposure to an alkaline environment and/or by acid injury [12, 44]. All these findings confirm the cytotoxic action both of ROS production and of acidic exposure leading to cells apoptosis. It is well known that vitD_3_ may prevent cell death through modulation of the interplay between apoptosis and autophagy. This effect is obtained by inhibiting superoxide anion generation, maintaining mitochondria function and cell viability, activating survival kinases, and inducing NO production [31]. Our results show that Grisù® alone is able to improve both cell viability and cell proliferation and in presence of vitD_3_ these effects are increased compared to control. In addition, after H_2_O_2_ or HCl exposure, Grisù® in association with vitD_3_ significantly reduced ROS production and decreased cell viability loss, suggesting that cell damage and cytotoxicity can be prevented by the combination of Grisù® with vitD_3_. These results suggest that Grisù® in combination with vitD_3_ may exert a better gastroprotective effect through an antioxidant pathway, inhibiting apoptosis and activating survival kinases. Such effect was stronger in preventing epithelial damage than what observed using other gastroprotective agents such as Gaviscon®, Maalox® or proton pump inhibitors.

Since GTL-16 is a cell line, further experiments were carried out in primary cells to validate the observed results. Cells isolated from gastric tissue were used. The results obtained from this new series of experiments have allowed us to significantly confirm beneficial effects of Grisù®, alone or in association with vitD_3_, to prevent the oxidative and acid injury.

In conclusion, this work demonstrates for the first time that vitD_3_ has a beneficial effect when combined with an alginate-based gastroprotector agent on gastric epithelial cells, joining the effects of a mechanical barrier with the modulation of intracellular pathways in order to maintain or restore the integrity of gastric epithelium.

## Conclusion

These in vitro results suggest that Grisù® in combination with vitD_3_ may exert a gastroprotective effect to maintain or restore the integrity of gastric epithelium through an antioxidant pathway, inhibiting apoptosis and activating survival kinases better than other gastroprotective agents.

However, further in vivo or research will be necessary in order to assess, with histological and immunocytochemical methods, the efficacy of Grisù® and vitD_3_ on gastric mucosa undergoing oxidative and/or acidic stress. The experimental data could thus provide a basis for the execution of clinical studies.

The combination of Grisù® with vitD_3_ significantly decreased the reactive oxygen species production and decreased cell viability loss, suggesting that cell damage and cytotoxicity can be reduced.

## Additional files

Additional file 1: Figure S1. Western Blot and densitometric analysis in GTL-16 cells. (DOCX 492 kb)
Additional file 2: Material and Methods; Results. Method: primary cell culture. Results: evaluation of Grisù® in combination with vitD_3_ on adhesion capacity (with 3 figures). (DOCX 338 kb)

## Abbreviation

BSA: Albumin from bovine serum
DMEM: Dulbecco’s Modified Eagle’s medium
FBS: Fetal bovine serum
GERD: Gastroesophageal reflux disease
MALT: Mucose-associated lymphoid tissue
MTT: 3-(4,5-dimethylthiazol-2-yl)-2,5-diphenyltetrazolium bromide
NO: Nitric oxide
PBS: Phosphate-buffered saline
PPI: Proton Pump inhibitor
ROS: Reactive oxygen species
VDR: Vitamin D receptor
vitD_3_: Vitamin D_3_
