# Supplementary material for: Role of vitamin D3 combined to alginates in preventing acid and oxidative injury in cultured gastric epithelial cells
Source: BMC Gastroenterol. 2016 Oct 7;16:127. doi: 10.1186/s12876-016-0543-z (PMC5054561; doi:10.1186/s12876-016-0543-z)
Supplement: Additional file 1: Figure S1. — Western Blot and densitometric analysis in GTL-16 cells. (DOCX 492 kb) [file 12876_2016_543_MOESM1_ESM.docx]

**Supplemental Figure**


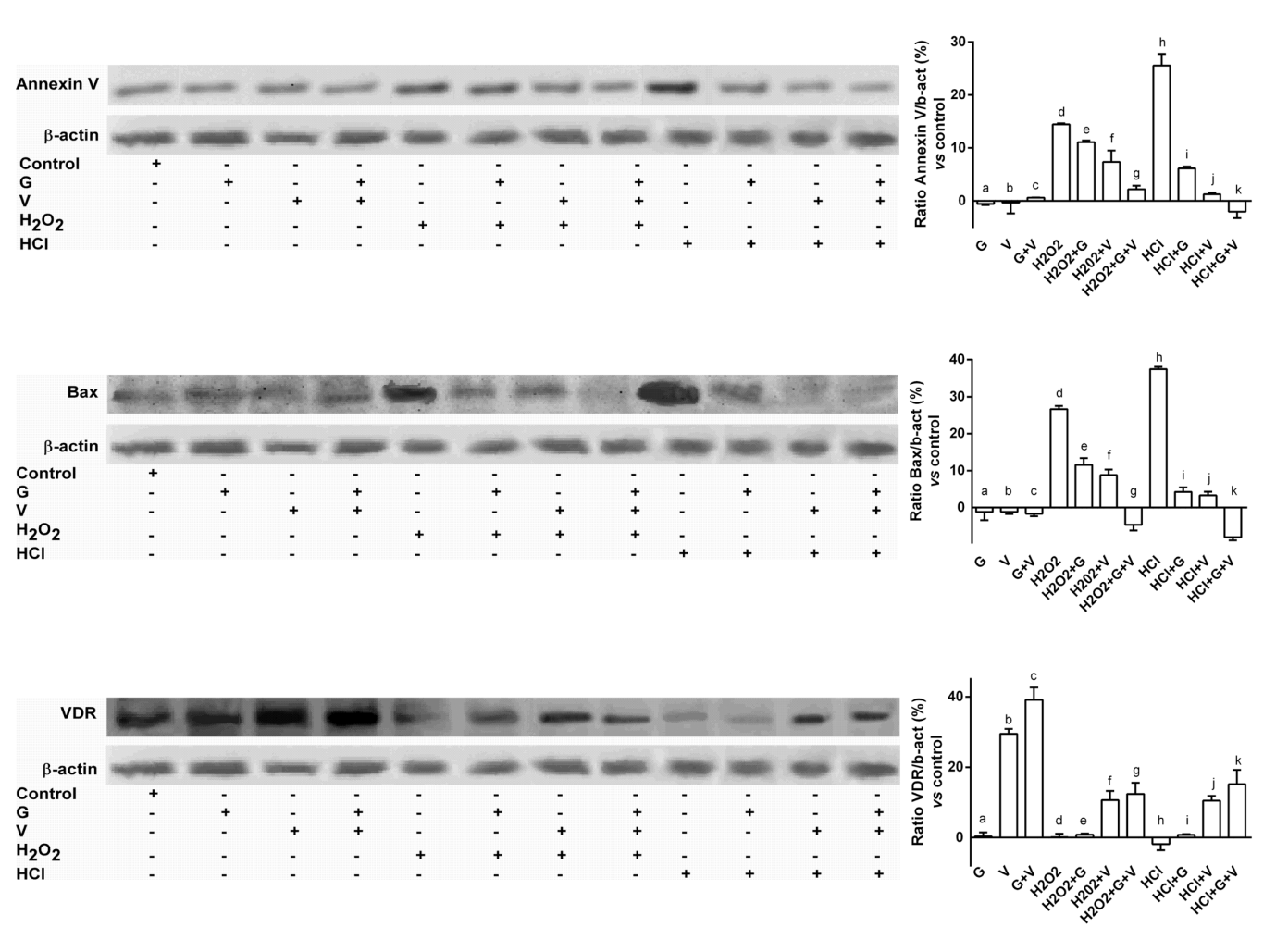


**S1** Western Blot and densitometric analysis in GTL-16 cells.

Western blot of protein extracts are analyzed by immunoblotting with specific antibodies against the indicated proteins with the relative densitometric analysis. The abbreviations are the same reported in the main manuscript. The images reported are an example of 5 biological replicates and the data are expressed as means ±(SD) (%). Annexin V *p*<0.05: d, e, f, h, i *vs* control (line 0%); e, f, g *vs* d; g *vs* k; i, j, k *vs* h. Bax *p*<0.05: d, e, f, g, h, i, j, k *vs* control (line 0%); i, j, k *vs* h; e, f, g *v*s d; g *vs* k. VDR *p*<0.05: b, c, f, g, j, k *vs* control (line 0%); f, g *vs* d; j, k *vs* h.
