# Supplementary material for: Role of vitamin D3 combined to alginates in preventing acid and oxidative injury in cultured gastric epithelial cells
Source: BMC Gastroenterol. 2016 Oct 7;16:127. doi: 10.1186/s12876-016-0543-z (PMC5054561; doi:10.1186/s12876-016-0543-z)
Supplement: Additional file 2: — Material and Methods; Results. Method: primary cell culture. Results: evaluation of Grisù® in combination with vitD3 on adhesion capacity (with 3 figures). (DOCX 338 kb) [file 12876_2016_543_MOESM2_ESM.docx]

**Supplemental data**

**Material and Methods.**

*Primary cell culture.*

Primary epithelial gastric cells, were obtained from the stomach of anaesthetized prepubescent pigs, following a previously described method for the isolation of human gastric epithelial cells [Smoot DT, Sewchand J, Young K, Desbordes BC, Allen CR, Naab T. “A method for establishing primary cultures of human gastric epithelial cells” Methods Cell Sci. 2000;22(2-3):133-6]. Briefly, the gastric cell were isolated from cut gastric tissue (8-10mm^3^) using enzymatic solution in agitation (collagenase/dispase solution, Sigma, Milan) for 60 min at 37°C. Then the suspension was centrifuged at 1500 rpm per 5 min at 4°C and then washed with PBS 1X. The pellet cells were then resuspend in complete medium (Ham’s F12 supplemented with 10% FBS, Sigma, Milan) on collagen coated dishes. The medium was changed each 3-4 days. The cells used for the experiments were obtained from passage 3 to passage 5. After the cells were used for MTT test, Crystal violet staining and Western Blot analysis placed at the same density used for GTL-16. The experiments were performed in the same conditions used with GTL-16.

**Results**

*Evaluation of Grisù*® *in combination with vitD_3_ on adhesion capacity.*

To confirm the data observed with GTL-16, Grisù® alone and combined with vitD_3_ were used to coat the multiwell plates before seeding the primary epithelial cells to study the number of adherent cells and the adhesion time (Figure S2.1). The adhesion time of primary cell was longer than in GTL-16 (starting from 10-12h). Using Grisù® this time was reduced, the cells started the adhesion after 15-30 min from seeding. This effect was more evident in presence of vitD_3_ (Figure S2.1), confirming the data observed with GTL-16.


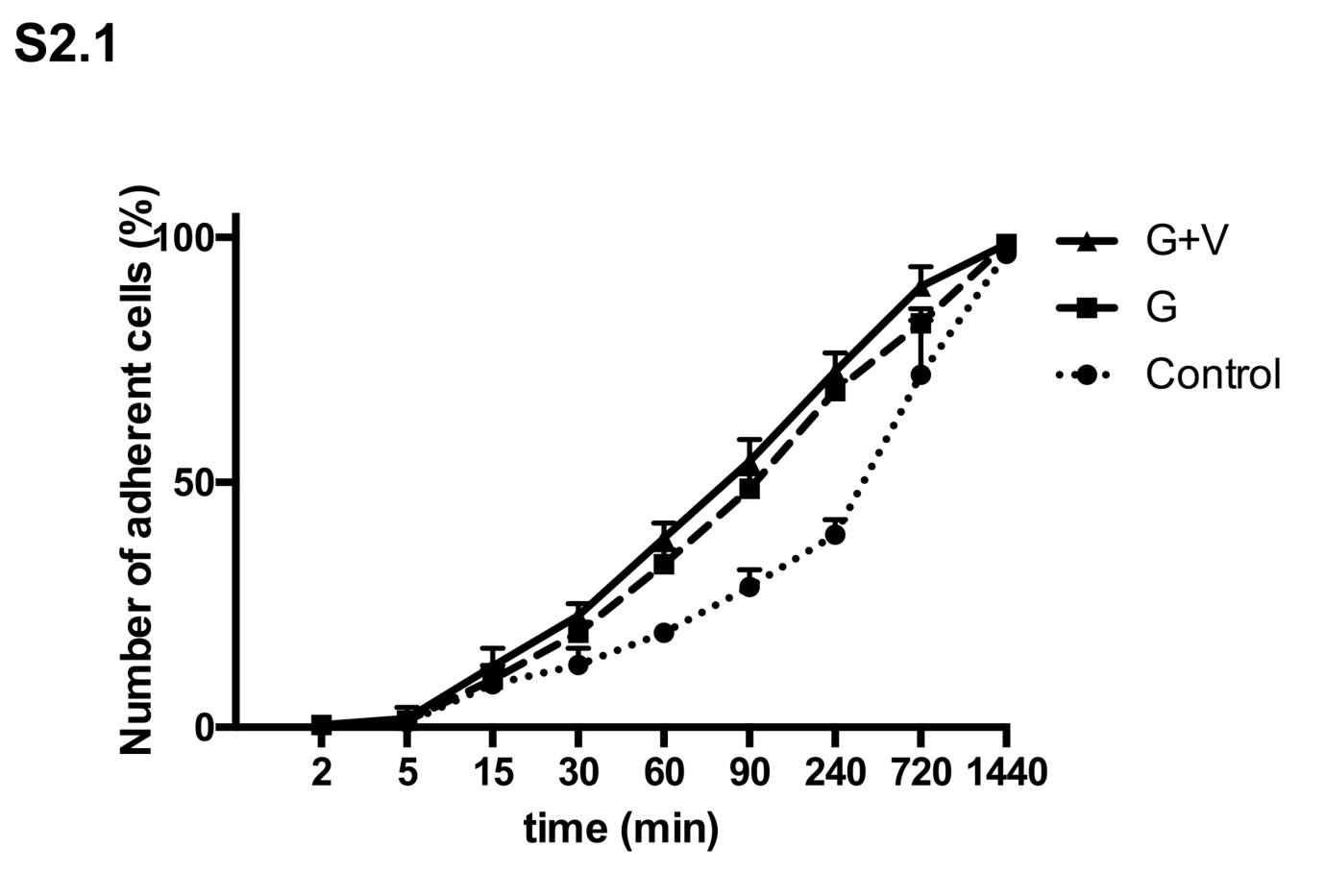


**S2.1.** Crystal violet staining.

Cell counting of adherent cells over time (at 2 min, 5 min, 15 min, 30 min, 60 min, 90 min, 240 min, 720 min and 1440 min) is reported. The ratio reports a mean±(SD) (%) of adherent cells counted in 12 different areas of 3 biological replicates. G=Grisù®; G+V= Grisù® combined with vitD_3_. Starting from 15 min samples were all significant *p*<0.05 *vs* control.

*Protective effects of Grisù*® *in combination with vitD_3_ in preventing damage caused by oxidative stress and acidity.*

To confirm effects on ROS production and cell viability, the experiments performed on GTL-16 were repeated using primary gastric epithelial cells. As reported in S2.2, in presence of oxidative stress and acidity, Grisù® alone and Grisù® combined with vitD_3_ were able to reduce ROS release and to enhance cell viability in similar manner to what observed in GTL-16, thus confirming the beneficial effects of vitD_3_ in gastroprotection. VitD_3_ amplifies the beneficial effects observed in presence with Grisù® alone (p<0.05).


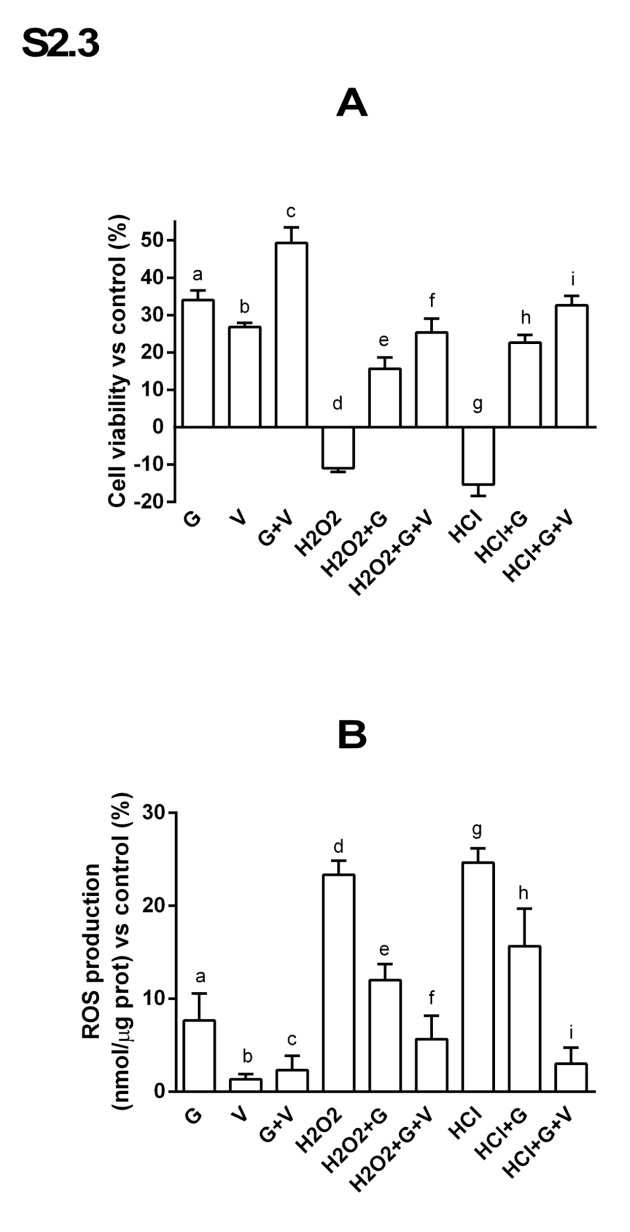


**S2.2.** Cell viability and ROS production in primary gastric cells.

Cells were treated in same conditions and with the same agents reported before, to analyze cell viability (A) and ROS production (B) during oxidative stress and acidity condition. G= Grisù®; V= vitD_3_; G+V= Grisù® combined with vitD_3_. Data reported are a mean±(SD) (%) of 3 biological replicates. In A *p*<0.05: a, b, c, d, e, f, g h, i *vs* control (line 0%); a, b, f, i *vs* c; e, f *vs* d; f *vs* e; h, i *vs* g; i *vs* h. In B *p*<0.05: a, d, e, f, g, h *vs* control (line 0%); c *vs* a; e, f *vs* d; f *vs* e; h, i *vs* g; i *vs* h.

In addition to what observed in GTL-16 on apoptosis and RISK pathway, primary epithelial gastric cells were also able to prevent apoptosis (Annexin V) and to induce the activation of survival signaling (ERK/MAPK) in presence of Grisù® alone (S2.3) These effects were more evident in the samples treated with Grisù® plus vitD_3_. These data support the results obtained in GTL-16 about the beneficial effects of the combination Grisù® and vitD_3_ to prevent the damage caused by oxidative stress and acidity. In addition, the presence of the modulation of VDR expression in these cells, confirmed the involvement of VDR receptor in all these effects.


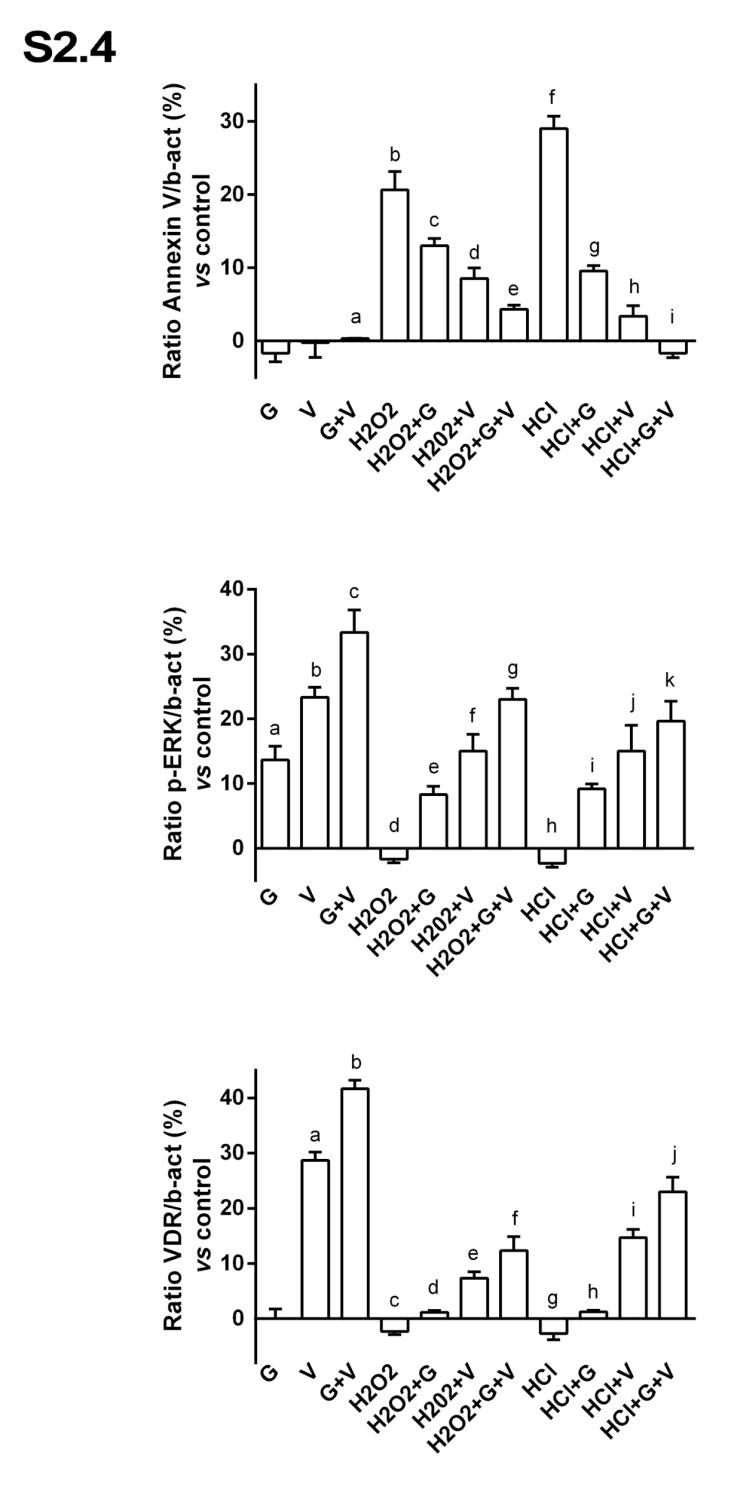


**S2.3.** Densitometric analysis of primary gastric cells.

The images reported are an example of 3 biological replicates. The abbreviations are the same reported previously. Densitometric analysis of Annexin V, ERK/MAPK and VDR receptor are normalized to control values and reported as means±(SD) (%) of 3 biological replicates. G= Grisù®; V= vitD_3_; G+V= Grisù® combined with vitD_3_. In A *p*<0.05: b, c, d, e, f, g, h *vs* control (line 0%); c, d, e *vs* b; e *vs* a; g, h, i *vs* f; g, h *vs* i. In B *p*<0.05: a, b, c, e, f, g, i, j, k *vs* control (line 0%); a, b, g, k *vs* c; e, f, g *vs* d; e, f *vs* g; i, j, k *vs* h; k *vs* i. In C *p*<0.05: a, b, e, f, i, j *vs* control (line 0%); d, e, f *vs* c; d, e *vs* f; h, i, j *vs* g; h, i *vs* j; f, j *vs* b.
